# Thin, Lightweight, and Highly Efficient Electromagnetic Interference Shielding Nanocomposites Composed of a π-Conjugated Block Copolymer Nanowire/Multiwalled Carbon Nanotube Bicontinuous Interpenetrating Network

**DOI:** 10.1021/acsomega.5c00452

**Published:** 2025-04-03

**Authors:** Yi-Huan Lee, Chian-Ling Wu, Ching-Wei Lai, Jun-Xing Huang

**Affiliations:** † Institute of Organic and Polymeric Materials, National Taipei University of Technology, Taipei 106344, Taiwan; ‡ Department of Molecular Science and Engineering, National Taipei University of Technology, Taipei 106344, Taiwan

## Abstract

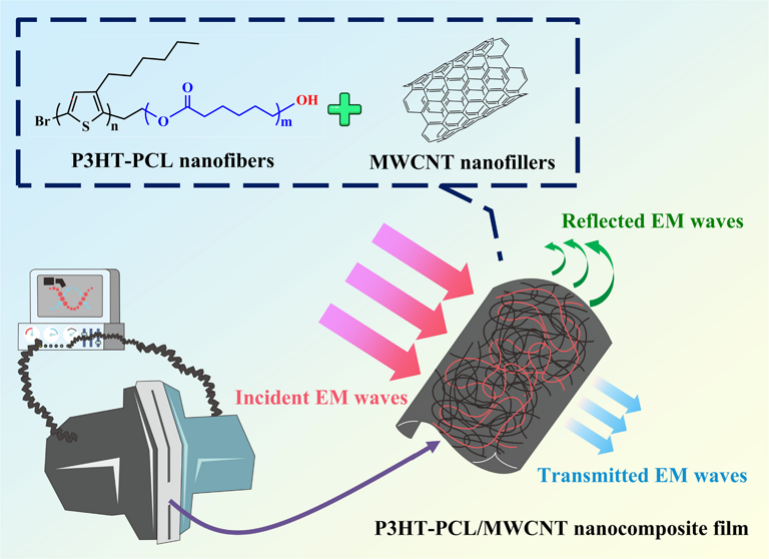

We developed high-performance electromagnetic interference (EMI) shielding composites by integrating a π-conjugated block polymer system with carbon nanofillers. First, a poly(3-hexylthiophene)–poly(ε-caprolactone) (P3HT–PCL) block copolymer system was synthesized through a combination of Grignard metathesis, tail modifications, and ring-opening polymerization. Controlling the solvent type and aging time resulted in the self-assembly of the P3HT–PCL chains into nanoscale fibers. When multiwalled carbon nanotubes (MWCNTs) were added to the P3HT–PCL solution, the π–π interactions between the thiophene rings of P3HT–PCL and the benzene units of the MWCNTs facilitated the dispersal of MWCNTs in the hybrid solution. Self-assembled P3HT–PCL chains prompted the formation of a nanofibrillar framework, which was beneficial to stabilize the overall structural distribution of the system, thereby synergistically forming a P3HT–PCL/MWCNT composite system with a bicontinuous interpenetrating network. The conductive skeleton formed by the effective connections of one-dimensional geometric MWCNTs made this composite system highly conductive and an excellent EMI shielding material. Moreover, the introduction of PCL helped overcome problems related to the rigid nature of P3HT, facilitating the manufacture of thin, lightweight, and high-performance shielding sheets. The developed composite film was 0.05 mm thick and provided an optimized EMI shielding effectiveness of up to 29.4 dB, effectively blocking nearly 99.89% of the incident waves. The results of this research provide an innovative and promising direction for the development of advanced EMI shielding materials.

## Introduction

Recently, π-conjugated polymers, including polythiophene, polyfluorene, and polypyrrole, have attracted widespread attention because of their unique conductive and optical properties. These features are attributed to their molecular structures, which involve alternating single and double bonds that allow internal charge carriers to move along the polymer chains.^1−3^ Polythiophene systems in particular are widely used in electronic technologies, including field-effect transistors, organic light-emitting diodes, organic solar cells, and sensors, owing to their crystallization capabilities and optoelectronic properties.^4−7^

As a consequence of the rapid development of communication technologies and electronics, individuals are increasingly being exposed to harmful electromagnetic (EM) waves in different frequency bands. EM waves affect the stability of electronic equipment and interfere with transmission in communication systems. These problems have necessitated the development of EM interference (EMI) shielding materials.^8,9^ Traditionally, EMI shielding systems have been fabricated using metals and have had excellent performance. Nevertheless, metal-based shielding systems have several disadvantages, such as heavy weight and susceptibility to rust. Also, they require expensive raw materials and extensive processing. EMI shielding composites composed of carbon nanofiller and polymer matrices have emerged as a promising alternative to traditional metal-based shielding systems because they are lightweight, mechanically flexible, corrosion-resistant, and cheaper to manufacture.^10−12^ For example, Ghosh et al. developed composites composed of acid-functionalized carbon nanofibers, carbon blacks, and a cocontinuous polymeric matrix. This material design enabled the system to exhibit a favorable absorption-dominated EMI shielding performance of 32.5 dB in X band.^13^ In addition to carbon nanomaterials, other nanofillers such as MXenes are also used in the development of EMI shielding composites.^14,15^ For example, Kang et al. fabricated a modified MXene sediment composite film system that exhibited an efficient shielding effectiveness of 15.8 dB in X band.^16^ Despite the gradual advancement of various fillers for EMI shielding, it is worth noting that carbon nanomaterials continue to receive widespread attention due to their low cost and mass production advantages.

The shielding effectiveness of EMI shielding composites depends largely on the type of carbon nanomaterial and its dispersion in the polymer matrix. Regardless of carbon nanomaterial used, strong π–π stacking interactions between carbon molecules of the nanomaterial often lead to severe aggregation, resulting in the uneven distribution of the nanomaterial molecules in the polymeric matrix, which diminishes the shielding performance.^17,18^ This problem can be overcome by using π-conjugated polythiophenes as structural templates because π-conjugated polythiophenes can effectively integrate with carbon nanomaterials through efficient π–π interactions between thiophene and benzene rings, facilitating the production of structurally homogeneous composites. Several polythiophene-based EMI shielding composite systems have been reported in the literature. Fang et al. integrated poly(3-hexylthiophene) (P3HT) and graphene nanosheets through a self-organization process for fabricating composite films with favorable hydrophobicity and EMI shielding performance.^19^ Nazir et al. developed a ferrocene-based polythiophene composite system, in which three carbon nanomaterials, namely, multiwalled carbon nanotube (MWCNT), reduced graphene oxide, and carbon black, could be uniformly incorporated into the polythiophene matrix. They explored the filler type effect on the conductive and shielding properties of the composite and emphasized the role of conductive network in shielding EM waves.^20^ Kumari et al. designed and constructed a conducting polymer composite system in which poly(3,4-ethylenedioxythiophene) and polyaniline, used as polymeric matrices, were combined with graphene. The heterogeneity of the components in their system led to efficient conductive channels and induced a prominent polarization effect. The formation of conductive structures and the polarization effect acted synergistically and improved EMI shielding performance.^21^

Despite continued advances in shielding performance, the rod-like configuration of polythiophene chains strongly hinders their molecular motions, causing polythiophenes to be too rigid and brittle. This, in turn, may substantially limit the flexibility and processability of the composites derived from polythiophene systems. This bottleneck can be overcome by integrating a flexible functional polymer with polythiophene to form a rod–coil block copolymer system.^22−26^ The introduction of soft chains can alleviate the inherently hard and brittle characteristics of polythiophene molecules, enhancing the toughness of the material and expanding its range of applications. Few studies have explored the use of polythiophene block copolymers in the fabrication of EMI shielding composites.

In this study, we developed high-performance EMI shielding composites by structurally integrating polythiophene block polymer nanofibers with MWCNTs. First, we synthesized a block copolymer system comprising P3HT and poly(ε-caprolactone) (PCL). The solvent type and aging time were adjusted to enable P3HT–PCL chains to self-assemble into nanofibers. MWCNTs were subsequently added to this self-assembly system. The added MWCNTs dispersed well in this hybrid solution, owing to the π–π interactions between the thiophene rings of P3HT–PCL and the benzene rings of the MWCNTs. The self-assembled nanowire network further stabilized the uniform distribution of the MWCNTs, resulting in the formation of a bicontinuous interpenetrating network of P3HT–PCL nanofibers and MWCNTs that conferred excellent electrical and shielding properties on the composite system. In addition to facilitating the dispersion of carbon nanomaterials, PCL improved the processability and flexibility of the composite system, which was conducive to the manufacture of thin, lightweight, and excellent shielding products. A 0.05 mm thick composite film with 80 wt % MWCNTs was produced. It exhibited an efficient shielding effect of 29.4 dB and was also flexible, effectively overcoming the shortcoming of rigidity ascribed to P3HT materials. We believe that this research will guide the future development of high-efficiency EMI shielding materials.

## Experimental Details

### Materials

2,5-Dibromo-3-hexylthiophene was synthesized using the Grignard metathesis method.^27^ Butylmagnesium chloride (2 M in tetrahydrofuran [THF]), [1,3-bis(diphenylphosphino)propane]dichloronickel(II), and stannous octoate were purchased from Sigma-Aldrich. Vinylmagnesium bromide (0.7 M in THF) was purchased from Thermo Scientific Chemicals. ε-Caprolactone was purchased from Alfa Aesar and distilled with calcium hydride before use. MWCNTs (BT1001M) with an average diameter of 10 nm and an average surface of 250 m^2^/g were purchased from LG Chem Ltd. THF was purchased from Acros and distilled with sodium before use. Other solvents and reagents were used as received without purification.

### Synthesis of P3HT-PCL Block Copolymer System

**Figure 1:**
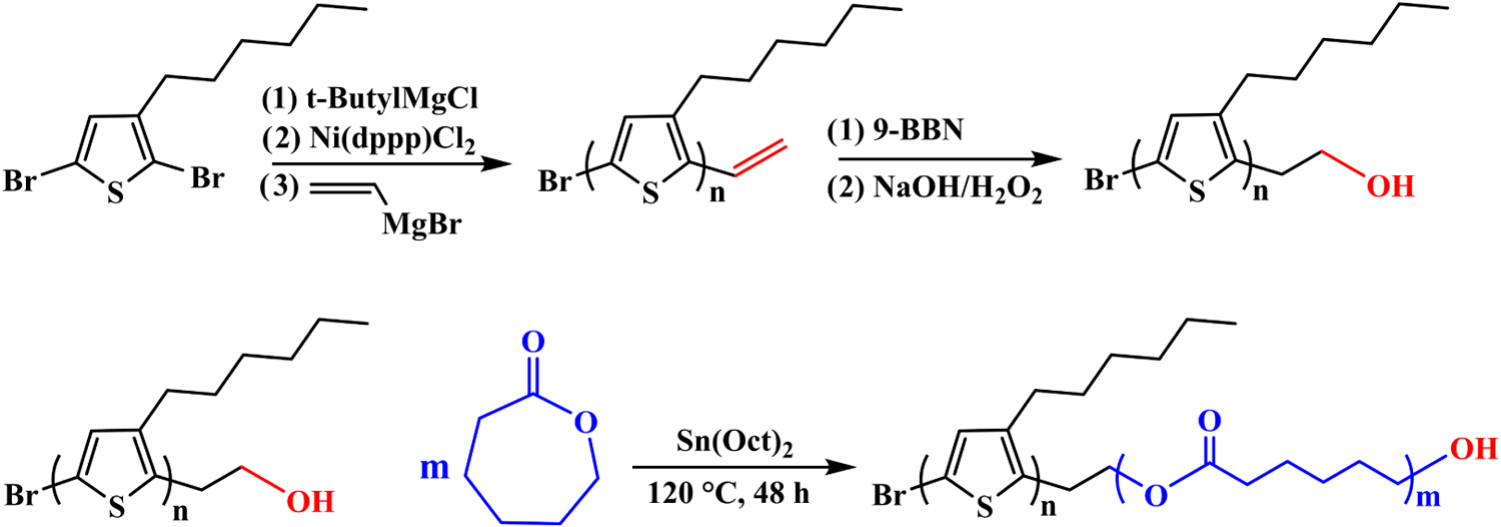
Reactions involved in the synthesis of the P3HT-PCL block copolymer system.

Figure 1 depicts the reactions involved in the synthesis of the P3HT–PCL block copolymer. 2,5-Dibromo-3-hexylthiophene (7 g, 21 mmol) and anhydrous THF (35 mL) were added into a 250 mL dry three-neck flask filled with nitrogen gas. Butylmagnesium chloride (11 mL, 22 mmol) was injected into the flask using a gastight syringe, and the mixture was vigorously stirred at room temperature. After 2 h, [1,3-bis(diphenylphosphino)propane]dichloronickel(II) (0.2 g, 0.369 mmol) was added to the flask, and the system was allowed to react at room temperature for 30 min. Next, vinylmagnesium bromide (7 mL, 7 mmol) was injected into the flask using a gastight syringe, and the system was allowed to react for an additional hour. The reaction was terminated using methanol, and the precipitated product was rinsed with acetone during Soxhlet extraction for preparing purified vinyl-terminated P3HT (P3HT–vinyl). The yield of P3HT-vinyl was 28%.

P3HT–vinyl (0.62 g) was then added to anhydrous THF (150 mL) in a 250 mL dry three-neck flask, and the mixture was stirred until dissolution. 9-Borabicyclo[3.3.1]nonane (10 mL, 5 mmol) was injected into the solution using a gastight syringe, and the flask was placed in an oil bath to allow the system to react at 40 °C for 24 h. A sodium hydroxide solution (4 mL, 6 M) was added to the flask, followed by vigorous agitation for 15 min. A 30% solution of hydrogen peroxide (4 mL) was further added to the flask, and the system was allowed to react at 40 °C for 24 h. The reaction was terminated using methanol, and the precipitated product was subjected to Soxhlet extraction for preparing purified hydroxy terminated P3HT (P3HT–OH). The yield of P3HT–OH was 90%.

P3HT–OH (0.55 g, 0.126 mmol), ε-caprolactone (0.53 g, 4.644 mmol), stannous octoate (65 mg, 0.161 mmol), and xylene (20 mL) were then placed in a 250 mL three-neck flask. After being freeze-pumped, the flask was placed in an oil bath at 120 °C for 48 h to facilitate polymerization. Finally, the reaction was terminated by using methanol, and the precipitated P3HT–PCL product was dried in a vacuum oven at 40 °C for 24 h. The yield of the P3HT–PCL block copolymer was 86%.

### Solution Aging of the P3HT–PCL Block Copolymer System

Two sample vials containing P3HT–PCL (10 mg) and THF (vial 1: 7 mL; vial 2: 5 ml) were prepared. The uniform dissolution of P3HT–PCL in THF was facilitated by heating the vials to 60 °C. Both vials were cooled to room temperature. The first solution (vial 1) was allowed to stand to facilitate aging. Acetone (2 mL) was added to the second solution (vial 2). The P3HT–PCL mixed solvent system in the second vial was incubated at room temperature for 48 h, followed by stirring to prepare a seed solution. This seed solution was allowed to stand to facilitate aging.

### Preparation of P3HT–PCL/MWCNT Composite Film

P3HT–PCL (10 mg) was uniformly dissolved in THF (5 mL) by heating the solution in a vial at 60 °C. The solution was then cooled to room temperature, and then, acetone (2 mL) was added to the solution. The solution was then subjected to a seed growth process by incubation at room temperature for 48 h. Subsequently, the solution was stirred uniformly, aged for 48 h, and ultrasonicated for 5 min. MWCNTs were added to the solution, and the solution was then uniformly stirred. After 24 h, the P3HT–PCL/MWCNT solution was cast onto a Teflon substrate at room temperature. After 48 h, the obtained film sample was further dried in a vacuum oven at 30 °C to ensure complete dryness. Finally, the completely dried film sample, with dimensions of 4.5 × 3.0 × 0.005 cm, was peeled off from the Teflon mold. In this study, a series of P3HT–PCL/MWCNT composite films was fabricated with MWCNT contents of 65, 70, 75, 80, and 85 wt %. The fabricated composite films are named HTCL*x*, where *x* represents MWCNT content. The thickness of the composite film system was measured using a Mitutoyo 293–240–30 outside micrometer.

### Proton Nuclear Magnetic Resonance (^1^H NMR)

The chemical structures of the synthesized P3HT–vinyl, P3HT–OH, and P3HT–PCL samples were characterized through ^1^H NMR spectroscopy performed by using a JNM-ECZS 400 MHz spectrometer at 25 °C. Deuterated chloroform served as the deuterated solvent for these measurements.

### Gel Permeation Chromatography (GPC)

GPC analyses were carried out using a Malvern Viscotek GPC system equipped with TM2500 and TM6000 M columns. THF served as the solvent for the measurements.

### Ultraviolet–Visible (UV–Vis) Spectroscopy

The UV–vis absorption behavior of the P3HT–PCL solution system was determined using a Jasco V-780 UV–vis spectrophotometer. The solutions to be measured were placed in a quartz cell, and the scanning wavelength ranged from 300 to 800 nm.

### Transmission Electron Microscopy (TEM)

TEM analyses were performed using a Hitachi H-7650 TEM system operated at an accelerating voltage of 75 kV. The TEM samples were prepared by dropping the polymer solutions onto carbon-coated copper grids and drying them at room temperature for 24 h. Bright-field images were recorded by using a Gatan model 782 charge-coupled device camera.

### Grazing-Incidence Wide-Angle X-ray Scattering (GIWAXS)

GIWAXS experiments were conducted at the 17A1 end-station of the National Synchrotron Radiation Research Center in Taiwan. The wavelength and incident angle of the X-rays were set to 1.321 Å and 0.2°, respectively. Silver behenate was used to calibrate the scattering vector q before the measurements.

### Measurement of Electrical Conductivity and EMI Shielding Performance

The conductivity values of the P3HT–MWCNT composites were measured using a Proskit MT-1210 multimeter. Additionally, the EMI shielding performance of the system in X band (8.2–12.4 GHz) was analyzed using a Keysight E5071C vector network analyzer equipped with waveguide holders.

## Result and Discussion

### Molecular Characterization of P3HT–PCL Block Copolymer

**Figure 2:**
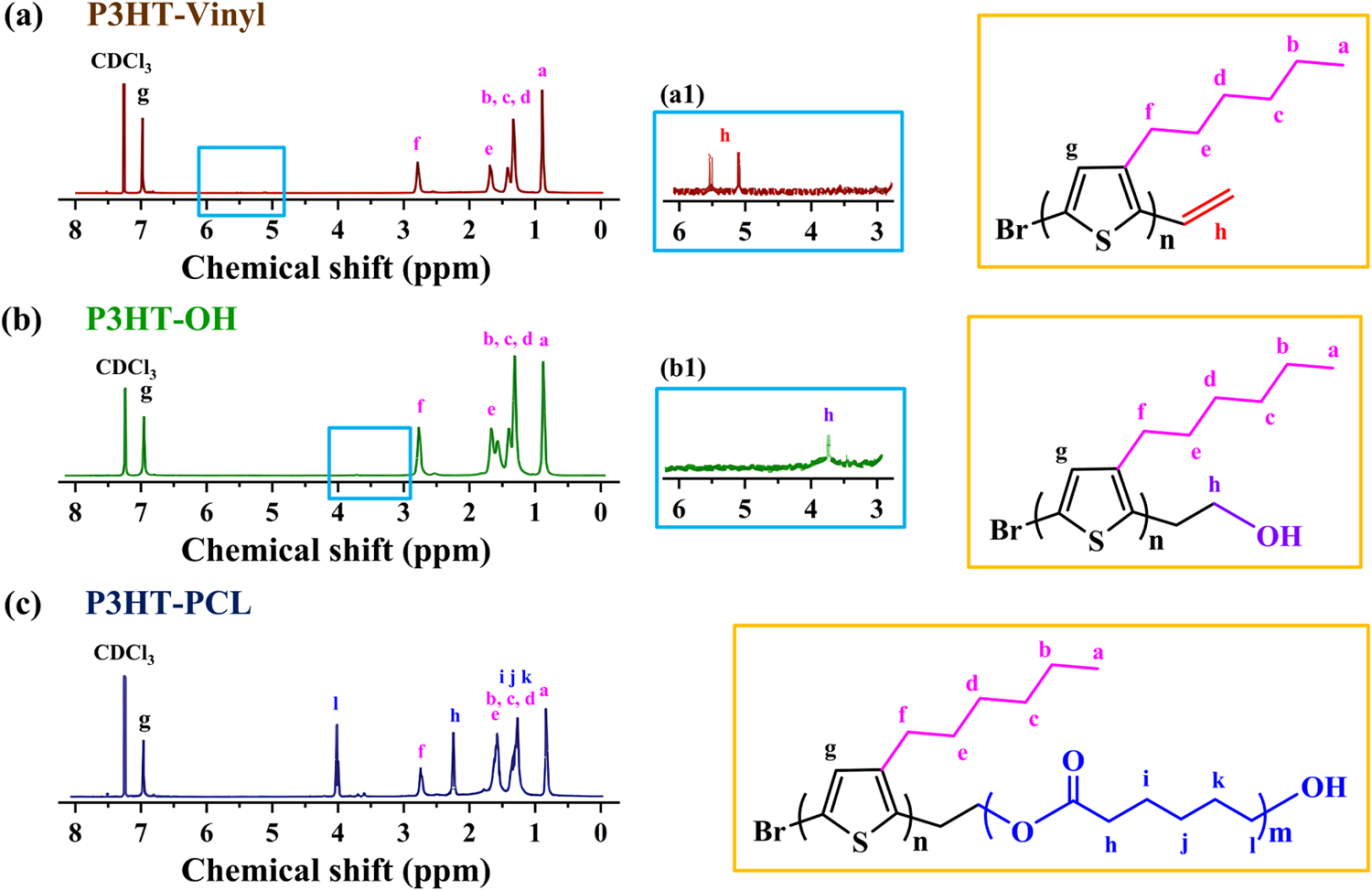
^1^H NMR spectra of (a) P3HT–vinyl, (b) P3HT–OH, and (c) P3HT–PCL block copolymer samples. (a1) and (b1) are the enlarged views of the local areas in (a) and (b), respectively.

P3HT–OH was used as a macroinitiator to initiate the ring-opening polymerization of ε-caprolactone in the synthesis of P3HT–PCL block copolymer. P3HT–OH was prepared by introducing vinylmagnesium bromide in the final stage of P3HT polymerization to modify the polymer chain termini to a vinyl group; this group was further transformed into alkylboride by reacting it with 9-borabicyclo[3.3.1]nonane. NaOH and H_2_O_2_ were added to the reaction system to convert the alkylboride into an α-hydroxyethyl group, which served as the reactive site for initiating the subsequent ring-opening polymerization of ε-caprolactone. Figure 2a presents the ^1^H NMR spectrum of the P3HT–vinyl sample, and Figure 2a1 shows an enlarged view of a local area. The peak at δ = 6.94 ppm was characteristic of the proton signal of the thiophene ring, and the peaks at δ = 0.87, 1.36, 1.66, and 2.78 ppm were attributed to methylene signals of the hexyl side chain attached to thiophene. Moreover, the resonance peaks at δ = 5.11 and 5.48 ppm were attributed to the resonance signals of the vinyl group, confirming that the vinyl functional group was successfully attached to the termini of P3HT. Figure 2b presents the ^1^H NMR spectrum of P3HT–OH, and Figure 2b1 presents an enlarged view of a local area. A resonance peak corresponding to the hydroxyethyl group was observed at δ = 3.75 ppm, whereas the original characteristic signals corresponding to the vinyl group disappeared, indicating that the vinyl group had been successfully replaced by the hydroxyl group. Figure 2c presents the ^1^H NMR spectrum of the P3HT–PCL block copolymer. In addition to the original P3HT signals, characteristic peaks attributed to PCL were observed at δ = 1.34, 1.63, 2.28, and 4.06. These results demonstrate that PCL was successfully synthesized by using P3HT–OH as a macroinitiator for the ring-opening polymerization reaction. The molecular weights of the synthesized P3HT–OH and P3HT–PCL composites were analyzed by using gel permeation chromatography. The GPC curves of the P3HT–OH macroinitiator and P3HT–PCL block copolymer are shown in Figure S1. The number-average molecular weights of P3HT–OH and P3HT–PCL were 9900 and 11 200 g/mol, respectively, and their polydispersity indexes were 1.39 and 1.57, respectively.

### Solution Self-Assembly of P3HT–PCL Block Copolymer

**Figure 3:**
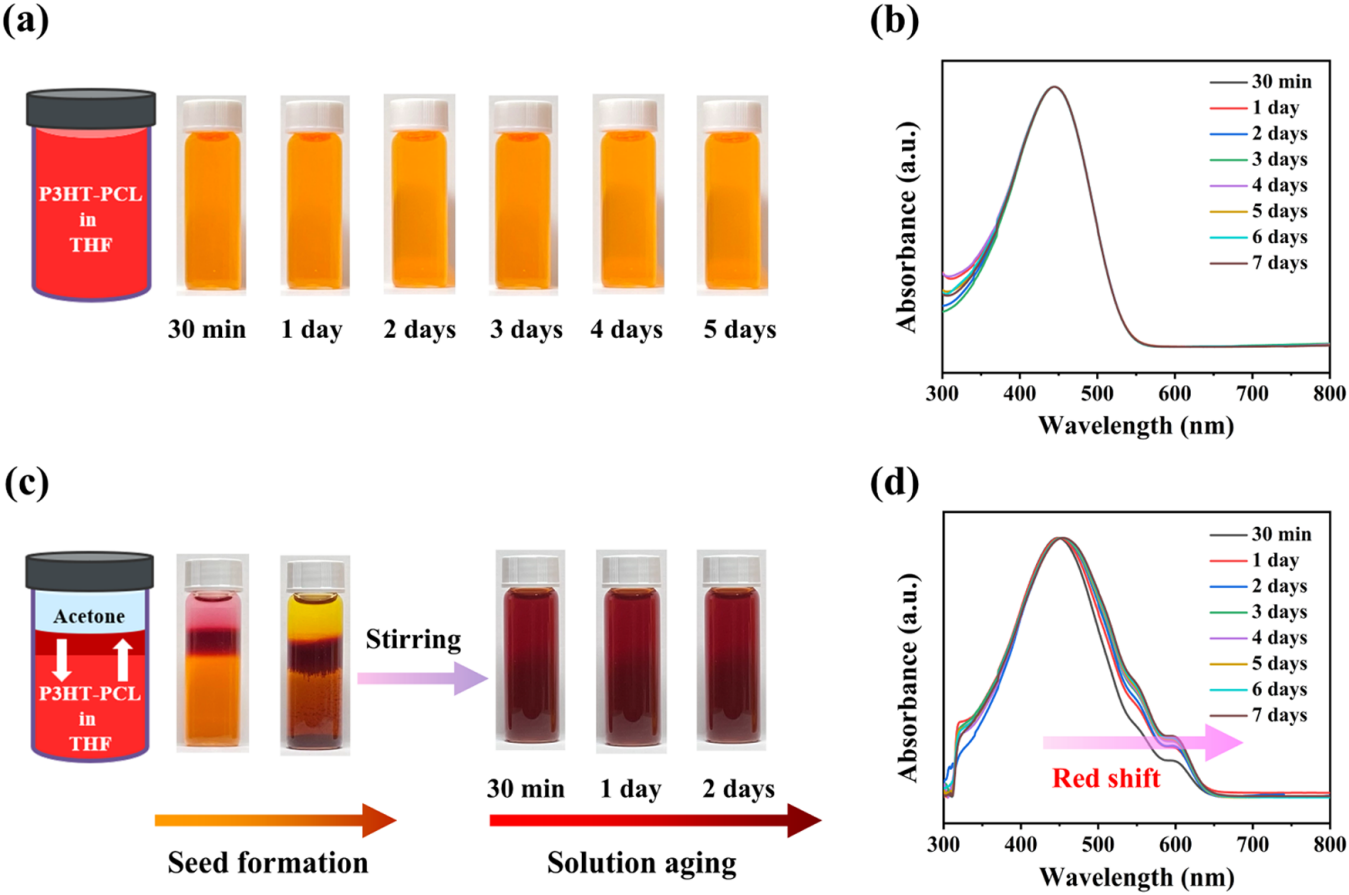
(a) Photographs of P3HT–PCL aged in THF. (b) UV–vis absorption spectra of P3HT–PCL aged in THF for different durations. (c) Photographs of P3HT–PCL aged in THF/acetone following seed growth. (d) UV–vis absorption spectra of P3HT–PCL aged in THF/acetone after seed solution preparation.

The solution self-assembly behavior of the P3HT-PCL block copolymer system was then explored. Figure 3a presents photographs of P3HT–PCL in THF (a good solvent for P3HT–PCL) corresponding to different aging times. The color of the solution, initially light orange, remained unchanged over time. The molecular behavior of the THF solution system was analyzed by UV–vis absorption spectroscopy. Despite different aging times, the UV–vis absorption spectra featured the same absorption maximum (λ_max_) at 445 nm, corresponding to the intrachain π–π* transition (Figure 3b).^28^ The combined results of visual investigation and UV–vis absorption spectroscopy indicated that P3HT–PCL molecules were dissolved uniformly in THF without any notable tendency to stack. The addition of acetone (a poor solvent for P3HT–PCL) into THF at a THF:acetone volumetric ratio of 5:2 decreased the solubility of P3HT–PCL. Consequently, the molecular chains gradually stacked together, and the color of the solution system progressively changed (Figure 3c). Owing to the density difference between THF and acetone, the interface between the two solvents could be maintained for several hours; this afforded better control over the diffusion and self-organization of molecules near the interface. As acetone contacted THF and the interface started to form, the solubility of the originally dissolved P3HT–PCL at the interface reduced, allowing the π-conjugated copolymer chains to self-organize into packed molecular chains; accordingly, the color of the solution changed from light orange to orange-red. The orange-red area gradually spread downward, and the color progressively deepened. After 48 h, the mixed solvent system was stirred uniformly and used as a seed crystal solution for further aging. Figure 3d illustrates the UV–vis absorption spectra of P3HT–PCL aged in THF–acetone after preparing the seed crystal solution. The λ_max_ gradually shifted from 446 to 455 nm. This red-shift indicates that the configuration of P3HT in P3HT–PCL progressively changed from the original coiled shape to a planar rod-like structure. During this transformation, the π-conjugated length of the P3HT segment was extended, synergistically leading to the progressive expansion of the electron delocalization range. This allowed electrons to move freely within a wider π-conjugated region, thereby reducing the absorption energy required for intrachain π–π* transitions and causing a red-shift in the absorption maxima. In addition to the red-shift, we observed two new spectral features at 552 and 601 nm, corresponding to the signals of the 0–1 and 0–0 transitions, respectively.^28,29^ The intensities of these two absorption peaks simultaneously increased over time. These spectral results indicate that the configurational transformation of the P3HT segment into a planar rod-like structure was synchronously conducive to establishing interchain π–π interactions that drove the self-assembly behavior of the P3HT–PCL system, resulting in the formation of highly ordered molecular packing.

**Figure 4:**
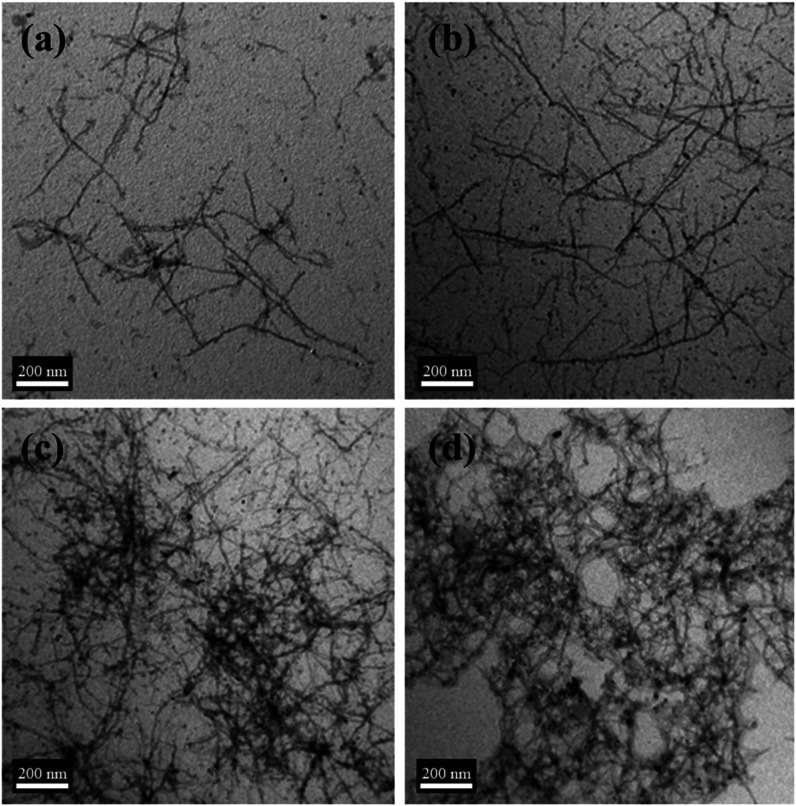
TEM images of P3HT–PCL aged in THF/acetone for (a) 30 min, (b) 2 days, (c) 4, and (d) 5 days.

**Figure 5:**
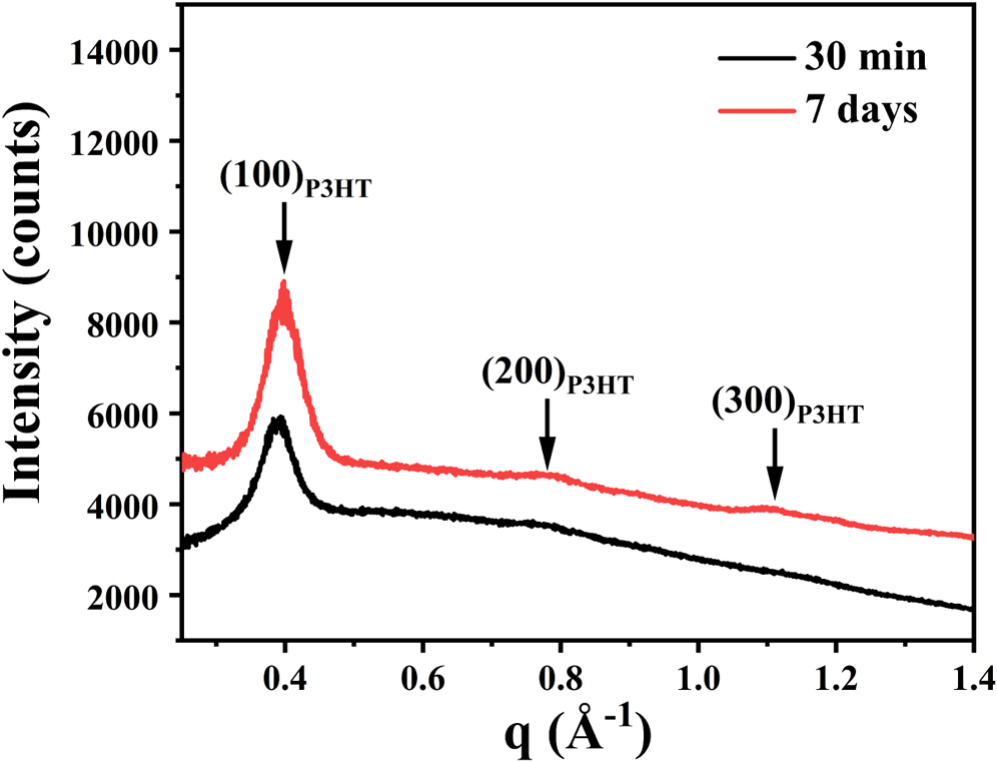
One-dimensional GIWAXS profiles of P3HT–PCL aged in THF/acetone for 30 min and 7 days.

The structures of the self-assembled P3HT–PCL molecules in THF/acetone were further characterized by using TEM. The micrograph of the P3HT–PCL sample aged for 30 min revealed a morphology of sparse nanofibers (Figure 4a). Both the number and extension length of the nanofibers increased significantly when the aging time was increased to 2 days (Figure 4b). The number and distribution density of the nanofibers further increased as the aging time was increased to 4 and 5 days (Figure 4c,d, respectively). The crystalline characteristics of the P3HT–PCL solution system aged for 30 min and 7 days were assessed using GIWAXS analyses. The two-dimensional scattering patterns are shown in Figure S2. Figure 5 depicts the one-dimensional scattering patterns. The GIWAXS profile of P3HT–PCL aged for 30 min displayed weak diffraction signals. In comparison, the GIWAXS profile of P3HT–PCL aged for 7 days displayed significantly enhanced diffraction peaks corresponding to P3HT. The increase in scattering signal intensity signifies an increase in the number of regularly packed P3HT–PCL molecules corresponding to the formation of nanofibers during aging. Overall, the results of visual investigations, UV–vis absorption spectroscopy, TEM, and GIWAXS analyses clearly demonstrate the ability of P3HT–PCL to self-assemble into regular nanofibers in an appropriate solvent environment. From the diameter measurements based on the TEM images (shown in Figure S3), the average diameters of MWCNTs and P3HT-PCL nanofibrils were 12.0 and 11.9 nm, respectively. In terms of material compatibility, the P3HT–PCL nanofibrillar network could be compatible with MWCNTs because of their similar nanoscale sizes and the π–π interactions between the π-conjugated thiophene rings of P3HT–PCL and the benzene rings of the MWCNT. The integration of P3HT–PCL nanofibers with MWCNTs is discussed in the following sections.

### P3HT–PCL/MWCNT Composites for EMI Shielding

**Figure 6:**
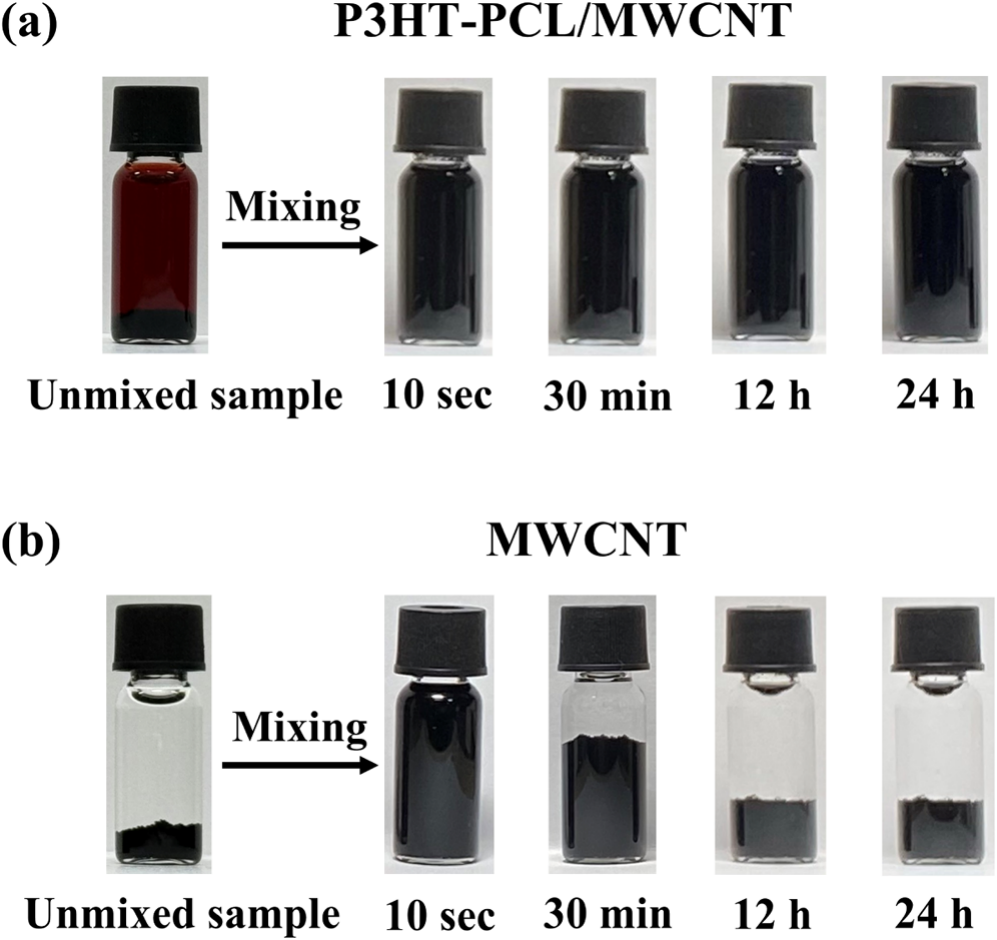
Dispersion of MWCNTs in (a) a P3HT-PCL nanofiber solution (in THF–acetone) and in (b) pure THF–acetone solvent.

**Figure 7:**
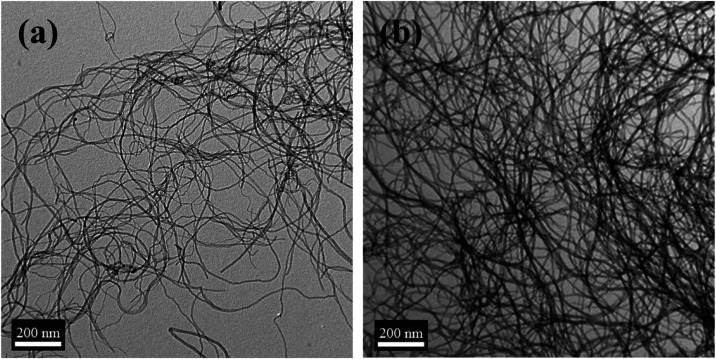
TEM micrographs of (a) pristine MWCNTs and (b) P3HT–PCL/MWCNT interpenetrating frameworks.

Figure 6a displays a series of photographs of the mixture sample prepared by adding MWCNTs into a P3HT–PCL solution system aged for 5 days in THF/acetone. The MWCNTs were uniformly mixed with the P3HT–PCL solution, and the uniformity of the MWCNT suspension was efficiently maintained, evidenced by the lack of sedimentation even after incubating the mixture for 24 h. By contrast, when MWCNTs were added to THF-acetone in the absence of P3HT–PCL, they rapidly sedimented (Figure 6b). We investigated the morphologies of pristine MWCNTs and the P3HT–PCL/MWCNT mixture using TEM. Pristine MWCNTs exhibited a one-dimensional tubular structure (Figure 7a). By contrast, the P3HT–PCL/MWCNT mixture exhibited an interpenetrating morphology comprising tubular MWCNTs and P3HT–PCL nanofibers (Figure 7b), implying that the self-assembled P3HT–PCL nanofibers effectively facilitated the uniform dispersion of MWCNTs in the hybrid system, resulting in a P3HT–PCL/MWCNT bicontinuous network. The formation of such highly compatible and stable dual frameworks was attributed to (1) the π–π interactions between the thiophene rings in P3HT–PCL and the benzene units in MWCNT and (2) the self-assembled P3HT–PCL nanofiber network that effectively stabilized the distribution of MWCNTs and prevented their aggregation.

**Figure 8:**
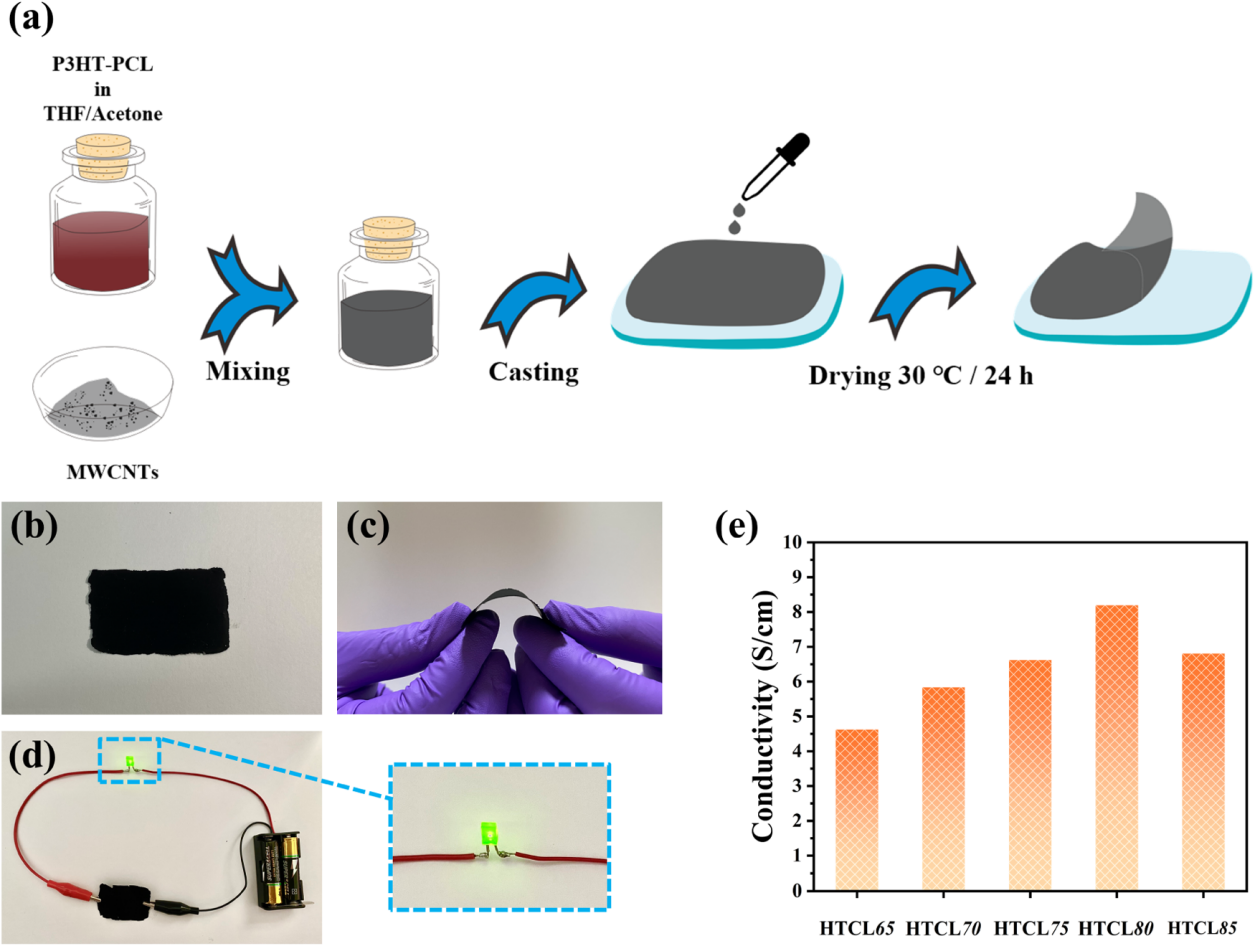
(a) Schematic of the manufacturing process of P3HT–PCL/MWCNT composite films. (b) Photograph of P3HT–PCL/MWCNT composite film. (c) Photograph of bent P3HT–PCL/MWCNT composite film. (d) Photograph of P3HT–PCL/MWCNT composite film used as a conductive material for lighting up light-emitting diode bulb. (e) Electrical conductivity values of P3HT–PCL/MWCNT composite films.

Figure 8a illustrates the process of preparing P3HT/PCL/MWCNT composite films. The prepared films had a dark appearance (Figure 8b). Moreover, the composite films exhibited flexibility owing to the introduction of the soft PCL block, making them suitable for use as flexible conductive materials to effectively light up a light-emitting diode bulb in a conductive loop (Figure 8c,d, respectively). We further fabricated a P3HT/MWCNT composite film with the same MWCNT content as HTCL80 and performed bending tests for the P3HT/MWCNT and HTCL80 composite films (the thickness of both films was 0.05 mm). It was observed that the P3HT–PCL/MWCNT composite film (HTCL80) was effectively bent repeatedly, while the P3HT/MWCNT composite film was broken during bending (Figure S4). These results clearly prove that the PCL soft block greatly improved the original rigid and brittle properties of P3HT and endowed the system with favorable flexibility and bendability. Figure 8e presents the electrical conductivity values of the composite films. When the MWCNT content increased from 65 to 80 wt %, the conductivity increased from 4.6 to 8.2 S/cm. When the MWCNT content further increased from 80 to 85 wt %, the conductivity of the composite system dropped to 6.8 S/cm. This decrease in conductivity could be due to the uneven dispersion of excess MWCNTs in the P3HT–PCL solution system, which may disrupt the distribution of the conductive network.

Next, we explored the EMI shielding performance of the P3HT–PCL/MWCNT composite system. Theoretically, the total EMI shielding effectiveness (*SE_T_*) of a material is a combination of reflection loss (*SE_R_*) and absorption loss (*SE_A_*), as shown in the following equation:^30^
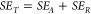
1The reflection *S*_11_ (or *S*_22_) and penetration *S*_12_ (or *S*_21_) scattering parameters of the system can be acquired using a vector network analyzer. Subsequently, these parameters can be used to calculate the power coefficients of reflection (*R*), transmission (*T*), and absorption (*A*), as shown in the following equations:^31^
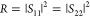
2
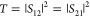
3
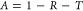
4The values of *SE_T_, SE*_*R*_, and *SE*_*A*_ can be calculated using the values of the power coefficients, as shown in the following equations:^31,32^
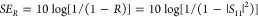
5
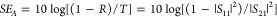
6
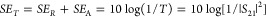
7

**Figure 9:**
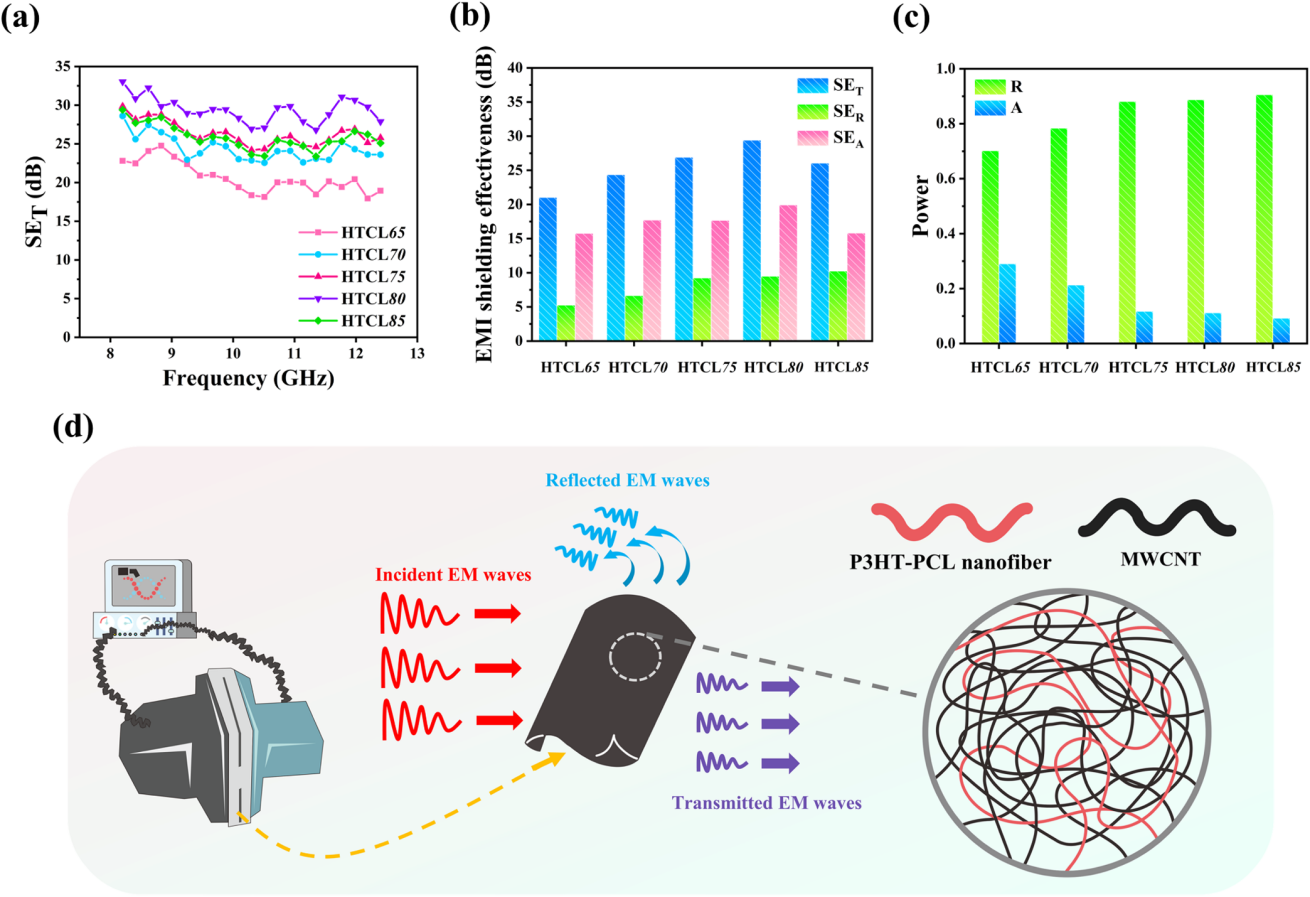
(a) *SE*_*T*_ curves in the X band of the P3HT–PCL/MWCNT composite system. (b) Average *SE*_*T*_, *SE*_*A*_, and *SE*_*R*_ values in the X band of the P3HT–PCL/MWCNT composite system. (c) *R, T*, and *A* values in the X band of the P3HT–PCL/MWCNT composite system. (d) Schematic of shielding mechanisms of the P3HT–PCL/MWCNT composite system.

Figure 9a illustrates the SE_*T*_ curves of the composite film system in X band, and Figure 9b presents the average SE_*T*_ values. The average SE_*T*_ values of HTCL65, HTCL70, HTCL75, and HTCL80 were 21.0, 24.4, 26.9, and 29.4 dB, respectively. The average *SE*_*T*_ of HTCL85 was 26.1 dB, which was lower than that of HTCL75. This performance decay at extremely high MWCNT loadings mirrored the conductivity results presented in Figure 8e, demonstrating that the excessive addition of MWCNTs disrupted the integrity of the conductive nanofiller network formed inside the P3HT–PCL system, resulting in a decrease in the ability to resist incoming EM waves. Figure 9b presents the mean *SE*_*R*_ and *SE*_*A*_ data in X band associated with the reflection and absorption contributions. The increasing and decreasing trends of *SE*_*R*_ and *SE*_*A*_ were consistent with those of *SE*_*T*_. These results indicate that the MWCNT content played a crucial role in the two different shielding mechanisms. A comprehensive comparison of the *SE*_*R*_ and *SE*_*A*_ data revealed that *SE*_*A*_ was higher than *SE*_*R*_ for each sample, implying that the dissipation of incident EM waves was mainly determined by absorption. Notably, this absorption dominance behavior was only observed within the composite material. The overall shielding mechanism of the system was further evaluated using the power coefficients.^33,34^Figure 9c presents the *R* and *A* values of the composite system. *R* was significantly larger than *A* for each composite film, indicating that the system as a whole was mainly based on a dominating reflection mechanism to block the incident EM radiation. Furthermore, the shielding performance of HTCL80 specimens with different thicknesses was further explored, and the summarized results are shown in Figure S5. It was observed that the *SE*_*T*_ values of HTCL80 films with thicknesses of 0.04, 0.05, and 0.07 mm were 27.7, 29.4, and 30.7 dB, respectively, indicating that increasing the thickness was beneficial to improve the EMI shielding effectiveness.

Figure 9d depicts a schematic illustration of the P3HT–PCL/MWCNT EMI shielding system. First, the incoming EM radiation was reflected at the composite surface because of a mismatch in impedance between the atmospheric medium and the conductive material system. Subsequently, the EM radiation that entered the interior of the composite material further interacted with the internal P3HT–PCL/MWCNT bicontinuous network driven by self-assembled P3HT–PCL chains. The conductive MWCNT network endowed the system with conduction loss ability. Moreover, interfacial polarization was simultaneously induced at the heterogeneous interfaces between the P3HT–PCL nanofibers and MWCNTs. A combination of conduction loss and polarization loss effects synergistically attenuated the energy of EM waves, giving the system excellent EMI shielding properties.^35^Table 1 further presents the EMI shielding performance information on this study and other reported carbon nanomaterials-based composites, in which our HTCL composite system reveals good competitiveness in the balance between shielding effectiveness and thinness, which is conducive to the application in thin, lightweight, and high-performance EMI shielding products.

**Table 1:** EMI Shielding Performance of Different Carbon Nanomaterial-Based Composite Film Systems

**material system**^a^	**thickness (mm)**	**EMI (dB)**	**frequency (GHz)**	**refs**
EMA/EOC/MWCNT	1	34.06	8.2–12.4	(36)
EMA/graphene	1	30	8.2–12.4	(37)
PVDF/MWCNT	1	35	8.2–12.4	(38)
PS/CNT	0.6	10.9	8.2–12.4	(39)
PVDF-HFP/MWCNT	0.15	25	8.2–12.4	(40)
PU/graphene/CNT	0.13	37.7	8.2–12.4	(41)
WPU/MWCNT	0.05	24	8.2–12.4	(42)
P3HT–PCL/MWCNT	0.05	29.4	8.2–12.4	this work

a EMA, EOC, PVDF, PS, PVDF-HFP, PU, and WPU are poly(ethylene-*co*-methyl acrylate), ethylene octene copolymer, polyvinylidene fluoride, polystyrene, poly(vinylidene fluoride-*co*-hexafluoropropylene), polyurethane, and waterborne polyurethane, respectively.

## Conclusions

A thin, lightweight, and high-performance EMI shielding composite system was developed in this study. First, a P3HT–PCL block copolymer was synthesized through a combination of Grignard metathesis, tail modifications, and ring-opening polymerization. The molecular structure of the block copolymer was characterized by using ^1^H NMR analysis. The self-assembly behavior of the P3HT–PCL system in solution was analyzed by adjusting the solvent type and aging time. When acetone was added to THF, the solubility of P3HT–PCL dramatically decreased at the interface of the two solvents, which allowed the molecular chains to stack into crystalline seeds. This solution was then subjected to aging. P3HT–PCL progressively self-assembled into an elongated nanofibrillar network in the solvent medium. Subsequently, MWCNTs were added to the self-assembly system. The π–π interactions between the thiophene rings of P3HT–PCL and the benzene units of MWCNT facilitated the dispersion of MWCNTs in the solution. The self-assembled P3HT–PCL nanofibrillar network prevented the aggregation of MWCNTs, which synergistically stabilized the hybrid system, resulting in the formation of a bicontinuous interpenetrating network of P3HT–PCL nanofibers and MWCNTs. This dual network structure endowed the composite system with excellent electrical and EMI shielding properties. The shielding effectiveness of a 0.05 mm thick HTCL80 composite film was 29.4 dB, implying that nearly 99.89% of the EM waves were shielded. Such an efficient blocking performance could be attributed to a combination of conductive MWCNT network driven by the self-assembled P3HT-PCL chains and the interfacial polarization between P3HT–PCL and MWCNT heteromaterials. These mechanisms synergistically allowed the incident EM waves to be effectively attenuated by the composite system. Moreover, the introduction of a soft PCL segment conferred flexibility on the system, overcoming the intrinsic rigid nature of P3HT. This research outlines a new direction in the development of high-performance EMI shielding materials.

## Supporting Information Available

GPC curves in Figure S1, two-dimensional GIWAXS patterns in Figure S2, TEM images with diameter measurement marks in Figure S3, bending testing in Figure S4, and shielding performance of composites with different thicknesses in Figure S5 (PDF)
