# Supplementary material for: Thin, Lightweight, and Highly Efficient Electromagnetic Interference Shielding Nanocomposites Composed of a π-Conjugated Block Copolymer Nanowire/Multiwalled Carbon Nanotube Bicontinuous Interpenetrating Network
Source: ACS Omega. 2025 Apr 3;10(14):14296–305. doi: 10.1021/acsomega.5c00452 (PMC12004186; doi:10.1021/acsomega.5c00452)
Supplement: Supplementary file 1 — ao5c00452_si_001.pdf [file ao5c00452_si_001.pdf]

Supporting Information:

Thin, Lightweight, and Highly Efficient Electromagnetic Interference Shielding Nanocomposites Composed of a π-Conjugated Block Copolymer Nanowire/Multiwalled Carbon Nanotube Bicontinuous Interpenetrating Network

Yi-Huan Lee,[1,2*] Chian-Ling Wu,[1] Ching-Wei Lai,[1] and Jun-Xing Huang[1]

[1] Institute of Organic and Polymeric Materials, National Taipei University of Technology, Taipei 106344, Taiwan

[2] Department of Molecular Science and Engineering, National Taipei University of Technology, Taipei 106344, Taiwan

* Corresponding author: Yi-Huan Lee, Associate Professor

Address: 1, Sec. 3, Zhongxiao E. Rd., National Taipei University of Technology, Taipei 106344, Taiwan

Phone No. 886-2-2771-2171#2410, Fax No. 886-2-2731-7174, Email: yihuanlee@mail.ntut.edu.tw

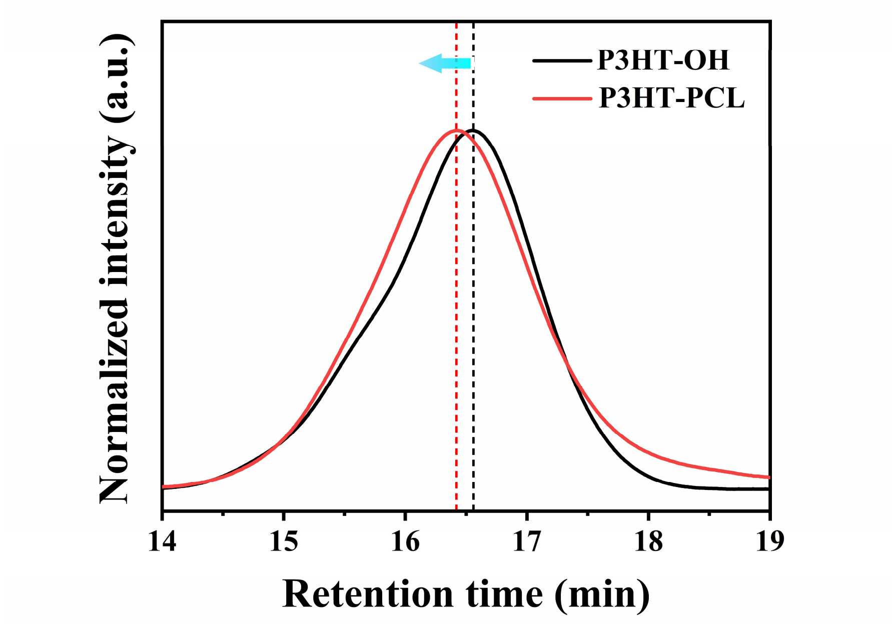

Figure S1. GPC curves of P3HT–OH and P3HT–PCL.

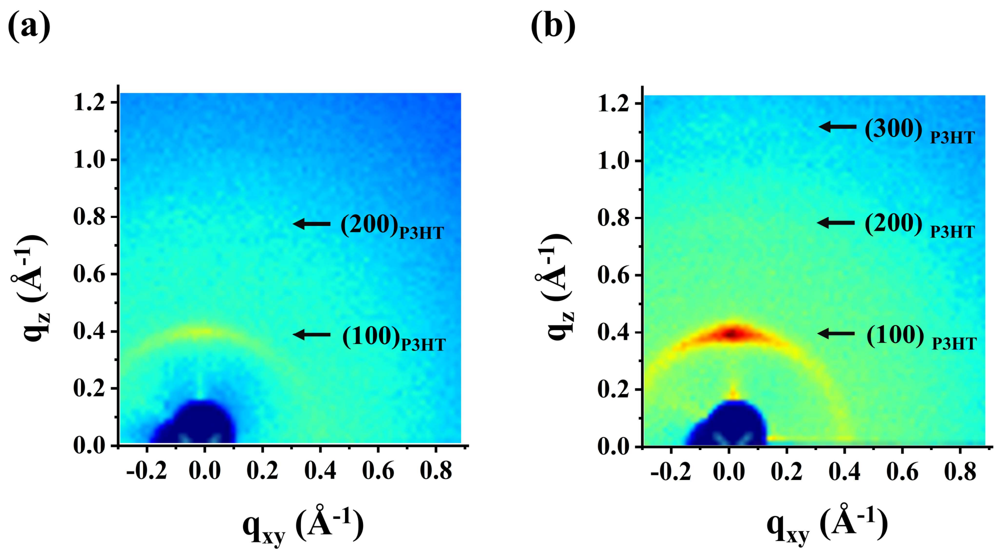

Figure S2. Two-dimensional GIWAXS patterns of P3HT–PCL block copolymers aged in THF/acetone for (a) 30 min and (b) 7 days.

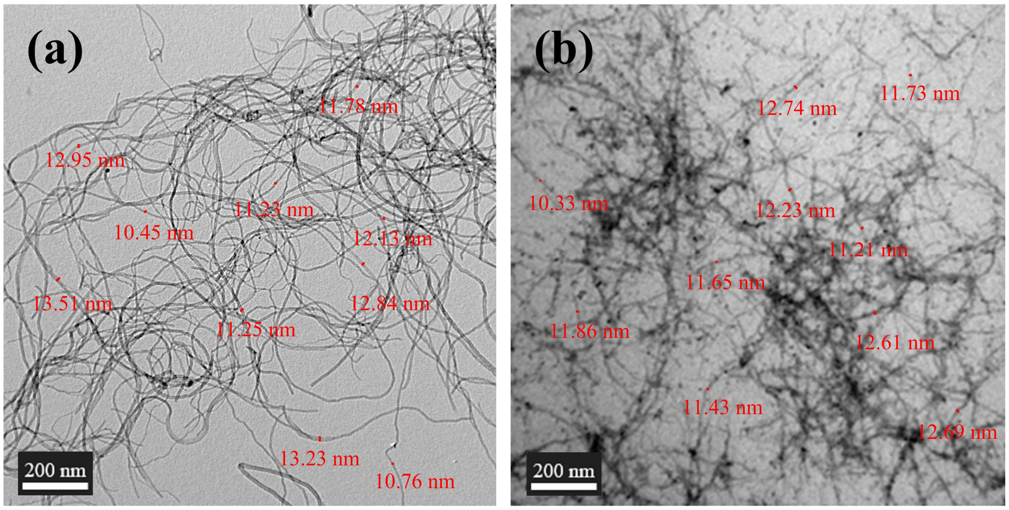

Figure S3. TEM images of (a) MWCNTs and (b) P3HT-PCL nanofibrils with diameter measurement marks.

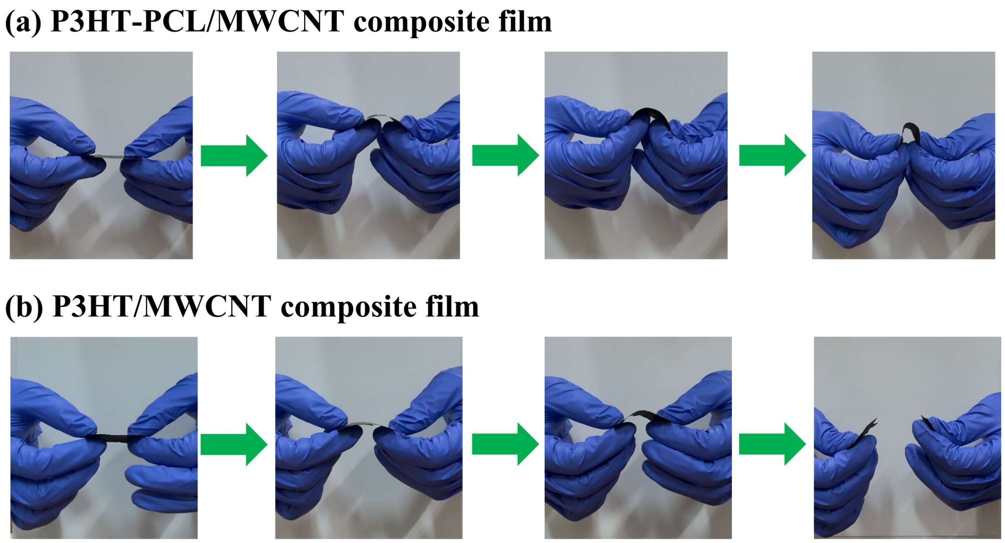

Figure S4. Bending testing for (a) P3HT–PCL/MWCNT (HTCL*80*) and (b) P3HT/MWCNT composite films with the same MWCNT content and thickness.

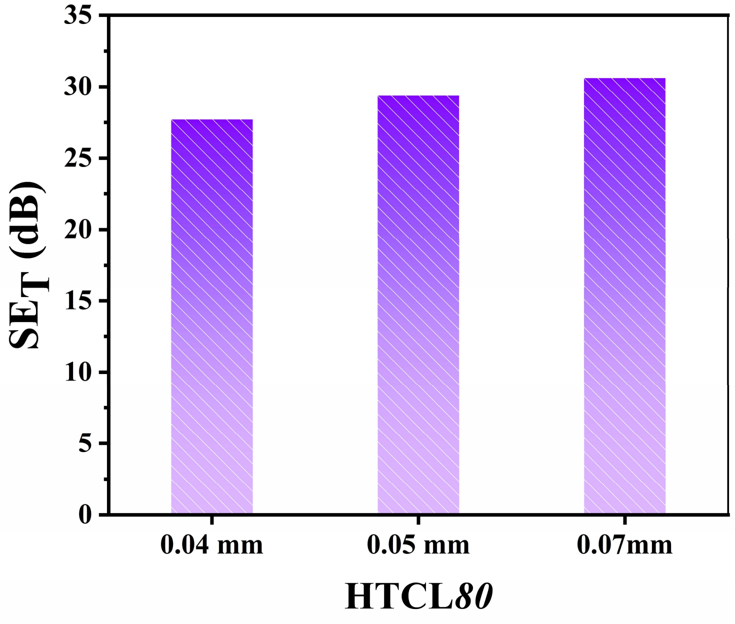

Figure S5. EMI shielding performance of HTCL*80* films with different thickness.
